# Hydrogel Models with Stiffness Gradients for Interrogating Pancreatic Cancer Cell Fate

**DOI:** 10.3390/bioengineering8030037

**Published:** 2021-03-13

**Authors:** Chun-Yi Chang, Chien-Chi Lin

**Affiliations:** 1 Weldon School of Biomedical Engineering, Purdue University, West Lafayette, IN 47907, USA; chang676@purdue.edu; 2 Department of Biomedical Engineering, Indiana University-Purdue University Indianapolis, Indianapolis, IN 46202, USA

**Keywords:** hydrogels, stiffness gradient, pancreatic cancer, durotaxis

## Abstract

Pancreatic ductal adenocarcinoma (PDAC) is the most common type of pancreatic cancer and has seen only modest improvements in patient survival rate over the past few decades. PDAC is highly aggressive and resistant to chemotherapy, owing to the presence of a dense and hypovascularized fibrotic tissue, which is composed of stromal cells and extracellular matrices. Increase deposition and crosslinking of matrices by stromal cells lead to a heterogeneous microenvironment that aids in PDAC development. In the past decade, various hydrogel-based, in vitro tumor models have been developed to mimic and recapitulate aspects of the tumor microenvironment in PDAC. Advances in hydrogel chemistry and engineering should provide a venue for discovering new insights regarding how matrix properties govern PDAC cell growth, migration, invasion, and drug resistance. These engineered hydrogels are ideal for understanding how variation in matrix properties contributes to the progressiveness of cancer cells, including durotaxis, the directional migration of cells in response to a stiffness gradient. This review surveys the various hydrogel-based, in vitro tumor models and the methods to generate gradient stiffness for studying migration and other cancer cell fate processes in PDAC.

## 1. Tumor Microenvironment in Pancreatic Ductal Adenocarcinoma

**Figure 1:**
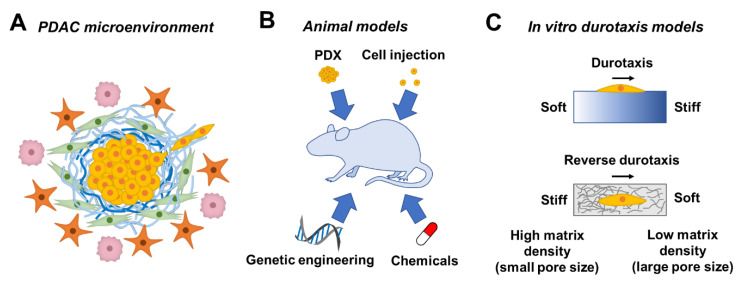
Schematic of (**A**) tumor microenvironment, (**B**) animal models used to study PDAC (PDX: patient-derived xenograft), and (**C**) in vitro durotaxis (or reverse durotaxis) models used to study cancer cell migration in response to stiffness gradient.

Pancreatic ductal adenocarcinoma (PDAC) is one of the most lethal cancers, with a 5-year survival rate of ~9% [1]. PDAC is characterized by a dense stroma (or desmoplasia) that accounts for ~ 90% of the total tumor volume [2]. Desmoplasia comprises various extracellular matrix (ECM) components, such as collagen, laminin, fibronectin, and hyaluronic acid (HA), as well as cellular components, including cancer cells, tumor-associated macrophages (TAMs), immune cells, and cancer-associated fibroblasts (CAFs). In contrast to highly vascularized pancreatic neuroendocrine tumors, stromal tissue in PDAC is poorly vascularized [3,4]. This is likely due to the compression of capillaries caused by the high interstitial fluid pressure of the stroma or due to the solid stress caused by the high cell and matrix density [5]. The highly dense desmoplasia also hinders therapeutic treatment efficacy as it acts as a physical barrier to block the chemotherapeutics from reaching the tumor cells [3]. While removing stromal tissue might improve drug penetration, this approach also removes the physical barrier restraining the cancer cells, leading to more aggressive PDAC [6]. These studies highlight the complicated roles of the tumor microenvironment (TME) in cancer cell fate (Figure 1A).

Among the stromal cells in the TME, CAFs have received significant attention due to their unique properties in supporting cancer cell growth and invasion [7,8,9]. CAFs are recognized as mesenchymal cells with elongated morphology and are negative for epithelial, endothelial, and leukocyte markers [10]. Most studies have established that CAFs express high levels of vimentin and α smooth muscle actin (α-SMA), whereas normal fibroblasts do not express α-SMA [11]. While CAFs are recognized as a major cellular component in the PDAC stromal tissue, they origin is still controversial, with some studies suggesting that CAFs arise from pancreatic stellate cells (PSCs) [12]. Upon activation through the secretion of multiple soluble factors by cancer cells and immune cells, such as transforming growth factor β (TGF-β), interleukin-1 (IL-1), IL-6, and IL-10, PSCs transition from a quiescent state to an activated state, which is characterized by the loss of lipid droplets and by the expression of α-SMA [12,13]. 

Activated CAFs promote tumor growth and metastasis through multiple mechanisms, such as ECM remodeling, secretion of cytokines, and immunosuppression [12]. Activation of CAFs also contributes to the stiffening of TME by matrix remodeling, including increased secretion of enzymes, excess deposition of ECM (e.g., HA) [14,15,16], and force-mediated matrix remodeling [17,18]. In particular, secretion of matrix metalloproteinase (MMP) by CAFs contributes to the matrix remodeling and cell invasion by forming “tracks” in the ECM, hence facilitating cancer cell migration [17]. To allow force-mediated matrix remodeling, CAFs form focal adhesion complexes on fibronectin through α5β1 integrin; thus, the increased contractility and traction force of CAFs can be applied to the matrix and form an aligned architecture that leads to the directional cancer cell migration [16]. For example, Labernadie et al. showed that CAFs can form E-cadherin/N-cadherin adhesion with A431 cancer cells, enabling CAFs to “drag” cancer cells while leading the invasion in a collagen matrix [19]. CAFs also express a variety of growth factors, cytokines, and chemokines that facilitate cancer cell invasion, including TGF-β1, leukemia inhibitory factor (LIF), growth arrest-specific protein 6 (GAS6), fibroblast growth factor 5 (FGF5), growth differentiation factor 15 (GDF15), and hepatocyte growth factor (HGF) [10]. In particular, TGF-β1 contributes to CAF activation through both paracrine and autocrine signaling [20,21]. TGF-β also exerts an immunosuppressive effect [22] and either suppresses tumorigenesis or promotes epithelial–mesenchymal transition (EMT) of the tumor depending on different stages during tumor development [23]. HGF has been shown to play a role in drug resistance through the upregulation of mitogen-activated protein kinase (MAPK) and phosphatidylinositol 3-kinase (PI3K)/protein kinase B (AKT) pathways [24]. In addition, IL-6 produced by CAFs not only has an immunosuppressive effect [25], but increases cholesterol uptake, which may be linked to the upregulated steroid biosynthesis and cholesterol metabolism by cancer cells [26]. Although the effects of the secreted cytokines and chemokines have been extensively studied, the effect of matrix stiffness on the expression of these cytokines and chemokines remains elusive and remains a topic of great interest.

The ECM components in PDAC stromal tissue include collagen, proteoglycans (e.g., HA), fibronectin, and laminin [27]. Among these matrices, collagen provides the basic framework of the ECM architecture and contributes to the tensile strength of tissue [28]. The homeostasis (e.g., degradation and deposition) of collagen is not well maintained in a cancer tissue [27]. In particular, elevated lysyl oxidase (LOX) expression by the primary tumor cells increases collagen crosslinking, leading to tissue fibrosis, and triggers pro-survival and proliferation signaling in other cancer cells [29]. 

HA, a glycosaminoglycan, is originally secreted in high molecular weight, i.e., ~2 MDa, by hyaluronan synthase 1 (HAS 1) and HAS 2, or ~200 kDa by HAS 3. HA can undergo degradation by hyaluronidase and become HA fragments (20–66 kDa) [30,31]. It has been reported that high-MW (>500 kDa) HA has anti-angiogenic and anti-inflammatory properties, whereas low-MW (20–200 kDa) HA exhibits angiogenic and pro-inflammatory properties [32]. For a more detailed review on how the molecular weight of HA affects cancer cells, readers are referred to the review by Price et al. [33]. HA is also increasingly accumulated in the TME as the tumor progresses [34]. A recent study showed that the accumulation of HA accounts for increased matrix (or tissue) stiffness in both in vitro and in vivo models [31]. The expression of cell surface receptors (e.g., CD44) to which HA binds is correlated with enhanced cancer cell proliferation, invasion, and drug resistance [35]. 

The accumulation of ECM affects not only cancer cell growth but also their invasion into the matrix, a dynamic process involving the interaction between cancer cells, stromal cells, and the ECM surrounding the tumor [36]. For example, the stiffness of the tumor tissue increases over a period of months to years. In particular, pancreatic tissue stiffness increases from a normal state of ~0.4 kPa to PDAC tissue of ~1.2 kPa (in Young’s modulus), as shown by the result of atomic force microscope (AFM) measurement (or from ~1 to ~4 kPa using the upper quartile to represent the stiffer region in the tissue) [37]. This physical property is caused by ECM remodeling, including enhanced deposition, alignment, and crosslinking [38]. The higher stiffness of the surrounding matrix, along with the abundance of collagen type I and fibronectin, increases the formation of invadopodia in the invading cancer cells, as well as the degradation of ECM [39]. 

Accumulating evidence has suggested that ECM remodeling could also be a result of the contraction force exerted by the cancer cells. The realigned and stress-stiffened ECM can in turn trigger the invasion of cancer cells from a spheroid, resulting in bidirectional interactions between the cancer cells and ECM [40,41]. The increase in ECM stiffness has also been shown to result in the malignant phenotype of mammary epithelial cells through integrin binding, which leads to activation of the PI3K pathway and Rac1 signaling [42]. Additionally, the hypoxia created in the TME by the rapidly proliferating cancer cells and a stiffened matrix has been shown to not only facilitate cancer cell migration through Notch signaling and Snail-1 upregulation but also increase collagen deposition and MMP production [43].

Solid tumors are known to exhibit an enhanced permeability and retention (EPR) effect, which is characterized by hypervasculature and a defective vasculature structure. From the perspective of drug targeting and accumulation, EPR may be beneficial and has been exploited for the delivery of chemotherapeutics [44]. However, the EPR effect is dependent on the type, location, and stage of tumor progression [45]. PDAC, despite its aggressiveness, is paradoxically characterized by an accumulation of ECM and poor vascularization [3,4]. Studies have shown that this hypovascularized region is limited to the juxta-tumoral compartment (<100 μm of tumor) in the PDAC TME, and higher density of blood vessels can be found in the pan-stromal compartment (or the region exterior to the juxta-tumoral compartment) [46]. Nguyen et al. proposed that cancer cells could invade and remove the vascular endothelium, a process that they termed “endothelial ablation”, and could potentially account for the hypovascularity in PDAC [47]. Additionally, it was reported that the microprojections of vasculature could be found in PDAC, which may explain the high glucose consumption of PDAC cells while surrounded by a thick layer of stroma and lack of vascularization [48]. Since PDAC is poorly vascularized, vascularization in hydrogel-based tumor models is not discussed in this review. Readers interested in 3D hydrogel-based approaches for modeling tumor vascularization are directed to prior reviews [49,50,51].

## 2. Animal Models for Studying PDAC Progression

Pancreatic cancer is associated with poor prognosis, mainly due to the difficulty of detecting the onset of tumor formation, the highly metastatic nature, high recurrence rate, and lack of typical symptoms. Therefore, a pancreatic tumor model can benefit clinical research by allowing observation since the onset of tumor formation, as well as different stages of the tumor, including metastasis. Over the past few decades, various animal models of pancreatic cancer have been developed (Figure 1B). This can be achieved through the administration of chemicals to rats to induce acinar cell carcinoma or acinar cell lesion [52,53,54,55]. Genetic engineering is another common approach to obtain pancreatic tumor models. A specific gene is typically transferred into a mouse or suppressed in a mouse through knock-in and knock-out techniques, respectively. Overexpression of the *Kras* gene through genetic engineering, for example, mimicked pancreatic tumorigenesis. Hingorani et al. reported that physiological levels of Kras^G12D^ induced ductal lesion that recapitulated the full spectrum of human pancreatic intraepithelial neoplasia (PanIN), precursors of PDAC [56]. Similarly, Aguirre et al. found that the combination of Kras^G12D^ activation and Ink4a/Arf suppression resulted in the early development of PanIN, which readily progressed to highly invasive and metastatic cancers [57]. 

In addition to chemical and genetic induction of mutation, an animal model of pancreatic cancer can also be achieved by injection of pancreatic cancer cells, either patient-derived or well-established cell lines, into nude mice or severe combined immunodeficient (SCID) mice, allowing the pancreatic cancer cells from human origin to develop into a tumor in vivo [58,59,60,61]. Similar to the former case, a patient-derived xenograft (PDX) model can be derived by transplanting a patient-derived tumor fragment into an SCID mouse [62]. Animal models demonstrated higher structural similarity to the tumor in patients, as compared to a 2D culture of pancreatic cancer cells or the co-culture of cancer cells and cancer-associated stromal cells. This makes animal models a valuable option for anti-cancer drug screening at a pre-clinical level [63,64]. Moreover, this heterogeneity of TME can be preserved using a PDX model. The high resemblance of histopathological and genetic characteristics of the xenograft to the parental tumor makes the PDX model more predictive of the clinical outcome [65]. For example, it has been found that the superior antitumor activity of combinational treatment of gemcitabine and *nab*-paclitaxel over gemcitabine or nab-paclitaxel alone was found in PDAC PDX models, which is in agreement with the phase 3 trial results [66,67]. However, animal models are expensive and may suffer from high variability in experimental output owing to the complexity of the in vivo environment and variations between animal species. More importantly, multiple factors/mechanisms come into play in TME, and it is difficult to decouple these factors/mechanism from one another using an animal model. Moreover, cell response and cell–cell/cell–ECM interaction remain elusive until the tumor tissue is resected, which makes it difficult to investigate whether cancer cells undergo durotaxis in vivo using an animal model. In this regard, hydrogels with a stiffness gradient are ideal for studying durotaxis or reverse durotaxis in vitro (Figure 1C). 

## 3. Mimicking a Stiffening Matrix Using Dynamic Hydrogels 

The stiffness of cell-laden hydrogels can be tuned through various methods, depending on the compositions and crosslinking mechanisms of the hydrogels (Table 1). For example, the stiffness of synthetic polyacrylamide (PA) hydrogels can be adjusted by tuning the concentrations of the monomer and crosslinker [68]. For synthetic polymers (e.g., poly(ethylene glycol) (PEG)) suitable for in situ cell encapsulation, hydrogel stiffness can be dynamically increased through diffusing additional photoinitiator and reactive polymers to allow for a secondary photocrosslinking [69]. Anseth and colleagues recently reported anthracene-modified PEG hydrogels that underwent [4 + 4] photodimerization under 365-nm irradiation [70]. The hydrogels were able to be stiffened multiple times in the presence of cells before reaching a stiffness plateau. Similarly, secondary photocrosslinking could be carried out using visible light [71,72]. Exploiting various photoreactive groups (e.g., acrylamidylpyrene and styrylpyren), Kalayci et al. developed hydrogels that could be stiffened based on [2 + 2] cycloaddition using visible light (wavelength: 410–490 nm) [71]. Our group has utilized flavin mononucleotide, a natural photosensitizer, to facilitate tyrosine dimerization in a hydrogel. Upon visible light (i.e., 440 nm) irradiation, the PEG-based hydrogel was effectively stiffened, with a more than two-fold increase in shear modulus [72].

As for chemically modified, natural polymers, secondary crosslinking can be used for gel stiffening once a primary network is formed. On-demand hydrogel stiffening can then be achieved using unreacted chemical moieties on the polymer chains. For example, Burdick and coworkers functionalized HA with methacrylates for forming a hydrogel network with dithiol crosslinker through an orthogonal Michael-type addition reaction [73,74]. Notably, the primary network was formed with an excess of methacrylate moieties, which were subsequently subjected to radical-mediated methacrylate homopolymerization, leading to dynamically stiffened hydrogels [75]. 

Gelatin-based hydrogels are increasingly used to mimic TME, owing to their inherent bioactivity and protease degradability. Gelatin hydrogels may be crosslinked via chain-polymerization via gelatin-methacryloyl (GelMA) or via orthogonal click chemistry, such as the thiol-norbornene photoclick reaction and tetrazine-norbornene click chemistry. In addition to homopolymerization of methacrylates, step-growth polymerization between mutually reactive macromers is increasingly being employed for the synthesis of dynamic hydrogels. For example, our group has synthesized gelatin-norbornene (GelNB), which is a diverse macromer susceptible to both thiol-norbornene and tetrazine-norbornene click chemistry [76,77,78]. With GelNB, gelatin-based hydrogels could be crosslinked via orthogonal thiol-norbornene click chemistry. GelNB can also be further modified with a secondary functional group (e.g., hydroxyphenylacetic acid, HPA), yielding a dual functional macromer, GelNB-HPA, susceptible to both light-mediated thiol-norbornene click reaction and tyrosinase-initiated di-HPA crosslinking for dynamic hydrogel stiffening [79]. Alternatively, a tyrosine-bearing peptide crosslinker (e.g., KCYGPQGIWGQYCK) was used for both thiol-norbornene photo-click reaction with PEGNB and tyrosinase-mediated dynamic crosslinking [80]. Taking advantage of the dual reactivity of the norbornene group towards thiol and tetrazine moieties, our group has recently reported orthogonal PEG-peptide thiol-norbornene hydrogels for culture and differentiation of human-induced pluripotent stem cells (hiPSCs). In particular, hiPSC-laden hydrogels were dynamically stiffened using tetrazine-modified macromers [81]. 

Another attractive method to dynamically stiffen, and even reversibly stiffen/soften, a hydrogel is through the host–guest supramolecular chemistry, such as affinity binding between β-cyclodextrin and adamantane or light-responsive azobenzene [82,83]. Once a primary hydrogel network is formed, the hydrogels were stiffened by adjusting the concentration of competing free cyclodextrin [84] or upon formation of host–guest complexes via the addition of PEG-adamantane [85]. Additionally, ionic interaction can be utilized for gel stiffening. For example, Stowers et al. created an alginate hydrogel that can be dynamically stiffened or softened through photothermal transition-triggered release of calcium or calcium chelator [86]. Poly(N-isopropylacrylamide) (PNIPAM) is known for its thermo-responsiveness and has been applied to dynamic hydrogel stiffening [87]. Upon heating above its lower critical solution temperature, PNIPAM transition into a dense hydrogel network through hydrophobic interaction, which, in turn, stiffens the hydrogels. Similar to a PA gel, while the polymer form is nontoxic, NIPAM monomer is cytotoxic, which can be present due to incomplete polymerization and limit the application [88].

**Table 1:** Chemistry for dynamic crosslinking of hydrogels to mimic tissue stiffening.

Mechanism	Material	Ref.
*Physical crosslinking*		
Supramolecular interactions	Azobenzene-HA + β-cyclodextrin-HA	[82]
Supramolecular interactions	o-Nitrobenzyl-methacrylate-HA + dithiothreitol	[83]
Supramolecular interactions	β-cyclodextrin-acrylamide + adamantane-acrylamide	[84]
Supramolecular interactions	Thiolated poly(vinyl alcohol) + PEG-allylether + β-cyclodextrin-allylether	[85]
Ionic crosslinking	Alginate	[86]
Temperature-responsive polymers	Polyisocyanide + poly(N-isopropylacrylamide)	[87]
*Chemical crosslinking*		
UV-based photocrosslinking	PEG-norbornene + thiol-bearing peptide	[69]
UV-based photocrosslinking	Methacrylated HA + dithiothreitol	[75]
UV-based photocrosslinking	PEG-anthracene	[70]
Visible light-based photocrosslinking	PEG-acrylamidylpyrene	[71]
Visible light-based photocrosslinking	PEG-norbornene + thiol/tyrosine-bearing peptide	[72]
Enzymatic crosslinking	Gelatin-norbornene-HPA + thiolated HA (or PEG4SH)	[79]
Enzymatic crosslinking	PEG-norbornene + thiol/tyrosine-bearing peptide	[80]
Click chemistry	PEG-norbornene + thiol-bearing peptide (or PEG4SH)	[81]

## 4. Stiffness Gradient in TME-Mimetic Hydrogels

### 4.1. Durotaxis in Cancer Cells

The stiffness gradient can be found in ECM under physiological or pathological conditions, such as embryonic development [89], myocardial infarction [90], and tumors [91]. It has been postulated that cell migration is affected by the presence of a stiffness gradient (i.e., durotaxis). Durotaxis, the directional cell movement from a soft substrate to a stiff substrate, has been observed and extensively studied in multiple cell types, such as fibroblasts, smooth muscle cells, stem cells, and cancer cells [92,93,94,95]. It is known that elevated ECM stiffness is concomitant with cancer cell invasion, and the force exerted by the cancer cells induces a heterogeneous alignment and stiffening on collagen fibers [40]. These findings imply the possible involvement of durotaxis in the PDAC cell invasion. Conversely, it has been reported that HT-1080 fibrosarcoma cells migrated from the stiffer region (360 Pa) to the softer region (100 Pa) within an MMP-degradable hydrogel, but not the reverse, a phenomenon referred to as “reverse durotaxis” (Figure 1C). The results also suggested that there was an upper limit of stiffness (560 Pa) for cell migration in the hydrogel, which was likely due to the inability of cells to degrade the matrix within the time frame [96]. It has been reported that invading cancer cells escaping from a cancer cell spheroid can exert force on the peripheral collagen matrix and induce strain-stiffening [40], which refers to the increased matrix stiffness caused by an applied strain [97]. Cells at the periphery of a tumor spheroid exerted high contractile force to the surrounding matrix once focal adhesions were formed. Additionally, migrating cancer cells can also strain-stiffen a collagen substrate [98]. Although cancer cell invasion in the PDAC TME might be the result of a synergistic effect of mechanical and chemical induction, it is likely that PDAC cells can exert force on the stiff peripheral ECM and strain-stiffen the peripheral ECM, leaving behind a stiffened and aligned fibrous ECM track for other PDAC cells to undergo durotaxis. However, in order for cancer cells to complete a metastatic dissemination, cancer cells must travel from the juxta-tumoral compartment to the softer pan-stromal compartment. Therefore, PDAC cells are also paradoxically speculated to undergo reverse durotaxis. Recent years have witnessed the development of various biomaterials with stiffness gradient for studying cancer cell migration (Table 2).

In addition to durotaxis observed for various cell types, cells can respond differently according to the substrate stiffness. For example, it was found that stiffness governs the lineage of stem cell differentiation [99]. It has also been shown that, when cultured on surface-functionalized PA gels with different stiffness levels, glioblastoma cells demonstrate substrate-stiffness-dependent morphology and behavior—glioblastoma cells demonstrated more prominent stress fibers and mature focal adhesion, as well as higher proliferation rate and motility, on a stiff (i.e., 119 kPa) PA gel or glass, while these expressions were diminished or abrogated on a compliant (i.e., 0.08 kPa) PA gel [100]. In the study by Lachowski et al., it was found that pancreatic stellate cells (PSCs) could return to a quiescent state, which was characterized by the decrease in α-SMA and vimentin expression, when the activated cells were cultured on Matrigel or a compliant (i.e., 1 kPa) surface-functionalized PA gel for 6 days [13]. In addition, increased stiffness in colon cancer resulted in the upregulation of TGF-β through the activin A signaling pathway by CAFs [101].

Both the stiffness range and the strength of the stiffness gradient are a crucial factor for studying cell durotaxis, as studies have shown that gradient strength or the steepness should exceed a threshold, the value of which is dependent on the stiffness of the initial position where a cell is located. That is, higher stiffness of the initial position stabilizes the cell adhesion and requires a higher gradient strength to activate durotaxis [102]. Using a PA substrate with a stiffness gradient below the threshold to induce durotaxis, Hadden et al. showed that nuclear translocation of Yes-associated protein (YAP), a known mechano-regulator, increases in a dose-dependent manner with respect to substrate stiffness in stem cells [103]. However, most of these studies were done using non-cancer cell lines. Durotaxis in cancer cells involving the interaction between cancer cells and CAFs is yet to be discovered. The stiffness range optimized for this 2D durotaxis is mostly 1–100 kPa. However, the stiffness range optimized for cell migration in a 3D matrix was reported to be much lower, since a stiffness of over 560 Pa was reported to hinder cancer cell migration [96].

**Table 2:** Examples of creating stiffness gradient in a hydrogel for cell studies (stiffness values listed are Young’s modulus, unless stated otherwise).

Material	Method	Gradient Range/Strength	Studied Cell Response	Ref.
*Natural polymers*				
Collagen	Compression on the material	1057–2305 kPa ~ 31.2 kPa/mm	Durotaxis	[104]
Collagen	Juxtaposition of soft and stiff gel	- -	Migration	[105]
Collagen	Juxtaposition of soft and stiff gel	50–217 Pa -	Migration	[106]
Matrigel	Patterned underlying substrate	- 0.27–0.37 N/cm *	Durotaxis	[107]
Fibrin	Strain-stiffening	- -	Orientation	[108]
*Modified natural polymers*				
Methacrylated gelatin	Photopolymerization	4–13 kPa 0.68 kPa/mm	Morphology, differentiation, and durotaxis	[109]
Methacrylated gelatin	Photopolymerization	23.7–1536.7 Pa (G’) -	Morphology and migration	[110]
Styrenated gelatin	Photopolymerization	2.2–83 kPa 40–1600 kPa/mm	Durotaxis	[102]
Methacrylated HA	Photopolymerization	0.5–1.5 kPa -	Spreading and differentiation	[111]
*Synthetic polymers*				
PA	Photopolymerization	1–80 kPa 0–40 kPa/mm	Morphology and durotaxis	[94]
PA	Photopolymerization	1–12 kPa 1/10/ ≥ 100 kPa/mm	Durotaxis	[95]
PA	Patterned underlying substrate	1–3.5 kPa -	Durotaxis	[112]
PA	Patterned underlying substrate	3–20 kPa 49.4–190.6 kPa/mm	Orientation and migration	[91]
PA	Gradient of diffusion rate	1–40 kPa 0.5–8.2 kPa/mm	Differentiation	[103]
PEGDM	Photopolymerization	2.05–6.11 kPa -	Phenotype maintenance and ECM deposition	[113]
PEGNB	Photopolymerization	100–360 Pa (G’)	Migration	[96]
PDMS	Thermal gradient	0.19–3.1 MPa 241 kPa/mm	Differentiation	[114]
PVA	Freeze–thaw cycle	1–24 kPa -	Adhesion, proliferation, and differentiation	[115]
PAH + PAA	Gradient of crosslinker	0.5–110 MPa -	Adhesion and proliferation	[116]

* Finite element simulation; Abbreviations: hyaluronic acid (HA); polyacrylamide (PA); polyethylene glycol dimethacrylate (PEGDM); polyethylene glycol norbornene (PEGNB); polydimethylsiloxane (PDMS); polyvinyl alcohol (PVA); poly (allylamine) hydrochloride (PAH); poly (acrylic acid) (PAA).

### 4.2. Creating Stiffness Gradient in Natural Matrices

**Figure 2:**
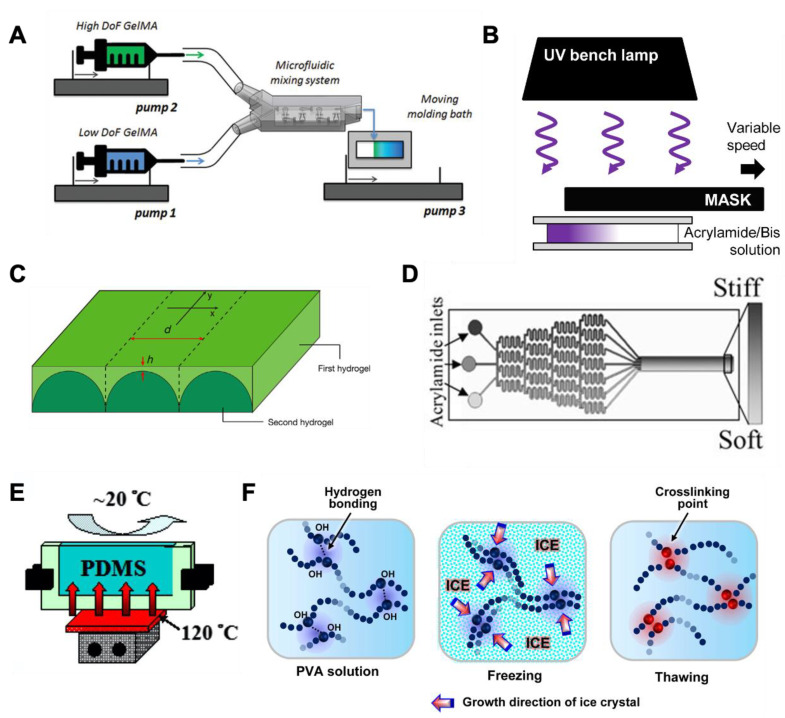
Hybrid and synthetic hydrogels used for creating stiffness gradient. (**A**) Gradient generation by syringe pumps. Reprinted with permission from [110]. (**B**) A moving photomask to create different degrees of photopolymerization. Reprinted with permission from [125]. (**C**) Microstructured hydrogel layer (bottom) and superficial gel layer (top) to create a mechanical interface with different stiffness levels. Reprinted with permission from [91]. (**D**) Microfluidic-based gradient generator was used for creating a gradient of bis-acrylamide that led to a gradient of crosslinking density upon UV irradiation. Reprinted with permission from [95]. (**E**) PDMS was cured with a temperature gradient, leading to a gradient of crosslinking. Reprinted with permission from [114]. (**F**) PVA was repeatedly dipped into liquid nitrogen to induce a gradient of physical crosslinking. Reprinted with permission from [115].

The use of collagen as a substrate to create a stiffness gradient for studying directional cell migration/durotaxis demonstrates high relevance in understanding the interaction between PDAC cells and the stiffness gradient in the TME (Figure 2A,B) [104,105]. For example, Hadjipanayi first fabricated a wedge-shaped collagen fibroblast-seeded construct, then compressed the construct into a thin gel sheet, thus creating a stiffness gradient across the construct [104]. They found that fibroblasts preferentially locate at the stiffer area of the gel, which is in agreement with the result using 2D synthetic polymeric material [93]. However, the ligand density and collagen fiber density could also be altered by the compression, which might have confounded the result. In another study, collagen gels of two different concentrations were juxtaposed, thus allowing the embedded cancer cells at the interface between the two gel formulations to experience a step gradient of stiffness. The MDA-MB-231 cells tended to migrate from the dense collagen matrix to the loose collagen matrix, indicating that the cells showed a tendency toward a more compliant 3D matrix of collagen [105]. This is in accordance with a more recent study by Sapudom et al. that suggested that pore size (i.e., cells migrate from small pores to large pores), rather than stiffness, is the factor that governs the directional cell migration of MDA-MB-231 in a heterogeneous collagen matrix [106]. Similarly, Puls et al. suggested that lower collagen fibril density led to increased EMT [117]. However, the factor of stiffness of the collagen matrix was inevitably confounded by that of the density of collagen fiber and cell adhesion ligands, and a higher collagen fiber density, in turn, entails smaller pore size, which could hinder cell migration. Although the effect of stiffness has been decoupled from the effect of the pore size of a collagen matrix [106], the stiffness range is relatively low, which is suitable for the study of breast cancer and might not apply to pancreatic cancer, which showed a higher stiffness range (i.e., Young’s modulus: 1–4 kPa) [37]. Similarly, Seo et al. suggested that the microarchitecture (e.g., pore size and fiber diameter) of the collagen matrix guides myofibroblast differentiation, and the effect is independent of the stiffness of the bulk collagen matrix [118].

While the non-linear stiffening of collagen at high stress (i.e., strain-stiffening) is stretch-dominated, at low stress, the stiffening is bend-dominated [119]. Collagen fibril bending stiffness is positively correlated with fibril diameter, and the increase in collagen fibril diameter has been reported to cause cell elongation and cell invasion by breast cancer cells [120,121]. On the contrary, despite the stiffness increase when stress above the linear/nonlinear threshold is exerted, collagen fiber alignment has been reported to cause a decrease in micro-scale stiffness, as measured by AFM [122]. This contradictory result of the strain-stiffening property could be due to the fact that the measurement is based on indentation into the surface of the aligned fibers, which is perpendicular to the alignment, and that the tip of the cantilever exerted a compressive force rather than a tensile force. Nevertheless, it is expected that the fiber alignment and the concomitant stiffness change can be tuned to create a stiffness gradient at micro-scale.

Other ECM-derived natural polymeric materials, including Matrigel and fibrin, have also been exploited for the same purpose [107,108]. Matrigel by itself is a compliant material with a homogeneous stiffness. Instead of creating a stiffness gradient within a Matrigel, Joaquin et al. deposited Matrigel on a wavy polydimethylsiloxane (PDMS) mold, which allowed the cells seeded on the Matrigel surface to sense the differential stiffness across the surface [107]. This was due to the higher stiffness of the PDMS compared to Matrigel, and the crest and trough of the wavy pattern imposed high and low “effective stiffness”, respectively, to the cells on top. Using a custom design, Kotlarchyk et al. induced torsion in a fibrin gel by rotating an embedded post. The shear strain across the gel is dependent on the position relative to the rotating post. The strain-stiffening property of fibrin allowed the transition of the strain gradient to a stiffness gradient [108].

### 4.3. Creating Stiffness Gradient in Hybrid and Synthetic Hydrogels

One drawback of natural polymers is the lack of tunability in the degree of crosslinking and the inability to control gel stiffness independently of the bioactive ligands in the scaffold. To this end, many natural polymers have been chemically modified to permit facile covalent crosslinking, such as gelatin-methacryloyl (GelMA) [109], styrenated gelatin [102], gelatin-norbornene (GelNB) [76,77], and HA-methacyloyl [111]. These modified natural macromers can be readily crosslinked into hydrogels via light- and radical-initiated photocrosslinking. The stiffness of these hydrogels can be tuned by changing the degree of functional group modification, by exposing the macromer solution via a photomask, or by adjusting the UV exposure time/intensity. These materials retain the ligands on natural polymers and allow excellent mechanical tunability. Although the majority of these studies were conducted using 2D substrates, a stiffness gradient in the 3D matrix can also be achieved, including the use of syringe pumps and a gradient mixer [123]. For example, Lavrentieva et al. mixed GelMA of two different degrees of functionalization in different proportions, thereby creating stiffness gradients for studying cell migration (Figure 2A) [110]. Overall, chemical modification of natural polymers adds versatility to and complements natural polymers for the generation of stiffness gradients [124].

Polyacrylamide (PA), a biologically inert material that can be mechanically tuned to reach a wide range of stiffness values (0.5–50 kPa) by adjusting the monomer and crosslinker concentration, has long been used for creating cell culture substrates among a variety of materials exploited for cell study [107]. Although polymerization can be achieved by simply mixing acrylamide, a bis-acrylamide crosslinker, and a radical initiator (e.g., ammonium persulfate, APS), the duration of the gel crosslinking and the final crosslinking density cannot be easily controlled. Furthermore, this process does not permit the spatiotemporal control of gel crosslinking and is not compatible with in situ cell encapsulation. To this end, photopolymerization has been applied to the fabrication of PA gels as it greatly reduces gelation time and provides spatiotemporal control of gelation. In this regard, stiffness gradients can be obtained within a PA gel by restricting the UV light transmission with a gradient (or patterned) photomask or a moving photomask (Figure 2B) [92,125], or by forming a layered structure with a stiff bottom layer and a compliant top layer, where the cells are seeded (Figure 2C) [91,103,107,112]. Within a critical distance between the cell monolayer and the underlying material, the “effective stiffness” experienced by the cells is higher when they are closer to the underlying stiffer material, and the stiffness decreases with increasing distance. Alternatively, the stiffness gradient in a PA gel can be created with the assistance of a microfluidic gradient mixer, which creates a gradient of monomer or crosslinker before UV light exposure [94,95]. For example, after the injection of a polymer solution (i.e., acrylamide solution with high- or low-concentration bis-acrylamide solution) into the inlet ports of a microfluidic gradient mixer, the polymer solution undergoes multiple splits and merges until the steady flow of the polymer solution with a bis-acrylamide gradient across the cross-section of the flow can be maintained (Figure 2D); this thus enables a gradient of crosslinking density once exposed to UV light [95]. However, the above-mentioned methods for establishing a well-defined and reproducible stiffness gradient often rely on advanced techniques (i.e., microfluidic system and photolithography) that require specialized facilities and equipment, which may not be easily accessible. Although PA hydrogel has been widely used for creating stiffness gradients, the possibility of the residual acrylamide to leach out and cause cytotoxicity has raised concerns about the use of PA for cell study, which led to other materials, such as PEG, being proposed as alternatives [113,126]. 

In addition to photopolymerization, Wang et al. described a method to create a stiffness gradient within a PDMS hydrogel sheet by inducing a thermal gradient in the material, leading to a gradient of crosslinking density and stiffness (Figure 2E) [114]. Kim et al. demonstrated that poly(vinyl alcohol) (PVA), a semi-crystalline polymer, can be physically crosslinked under freeze–thaw cycles, which was achieved by repeatedly dipping the PVA solution into and lifting it out of liquid nitrogen at a controlled speed (Figure 2F) [115]. Similarly, a gradient of amide bond formation was induced in a polyelectrolyte multilayer of poly(allylamine hydrochloride) and poly(acrylic acid) by dipping the multilayer film into a solution of 1-ethyl-3-(3-dimethylaminopropyl) carbodiimide (EDC) [116]. Synthetic materials provide a wide range of stiffness and gradient strength values for studying cell responses to the stiffness gradient of a substrate, and their bio-inertness prevents the interference of direct biological signaling, but the lack of ECM components often requires surface functionalization. For the same reason, hydrogels made with synthetic polymers require both cell adhesion ligands and MMP-degradable peptides to allow 3D cell migration, which limits their application [96].

## 5. Conclusions and Future Directions

TME is a complex milieu where cell–cell interactions and cell–ECM interactions collectively govern cancer progression. To enable a mechanistic understanding of cancer cell fate, considerable efforts have been dedicated to designing bioengineered materials and methods for studying these interactions. Among the stromal cells in PDAC TME, CAF plays a crucial role in assisting tumor growth and EMT. This is achieved through the secretion of abundant cytokines and chemokines by CAFs [10]. In addition, the matrix remodeling by CAFs and reported E-cadherin/N-cadherin adhesion between CAFs and cancer cells both facilitate cancer cell invasion [19]. ECM stiffness in TME is known to increase through the excessive ECM deposition, additional crosslinking, and stress-stiffening. Although a dense stroma is reported to restrain PDAC, PDAC cells can adapt to and circumvent this restraint through alternative nutrient uptake and potentially through microprojection of vasculature [48,127,128]. The stiffness increase in ECM also triggers the invasion of tumor cells. However, the cause of tumor cell invasion is confounded by multiple factors, including chemical and mechanical signals. Although durotaxis of multiple cell types has been studied in vitro [92,93,94,95], whether cells are capable of durotaxis in vivo remains largely unknown. There have been contradicting theories as to whether cancer cells undergo durotaxis or reverse durotaxis in TME.

Tumor models using hydrogel as a 3D matrix have been widely adopted since hydrogels have the capability to recapitulate multiple aspects of a TME. This provides a 3D microenvironment that mimics the natural ECM-like architecture and provides high tunability of the physical and chemical properties, such as stiffness and cell adhesion sites. Therefore, a hydrogel-based PDAC tumor model with a stiffness gradient may provide new insight into how PDAC cells respond to stiffness changes in TME, as most 2D durotaxis studies have suggested a stiffness range much higher than that of 3D cell migration studies. To allow cell migration study within a hydrogel, cells should be encapsulated in a primary gel network amenable to cell-mediated matrix remodeling and user-induced dynamic and gradient stiffening. This can be achieved through various possible approaches, one of which is through the diffusion of macromers with reactive moieties into the primary gel network, where additional crosslinking can take place. Another viable option is to create a stiffness gradient using temporal secondary photocrosslinking within the cell-laden network. With the incorporation of relevant cellular and ECM components, the tumor models can recapitulate multiple aspects of PDAC TME and provide a powerful tool for studying PDAC cell growth, invasion, EMT, and drug resistance.

## Abbreviations

PDAC	Pancreatic ductal adenocarcinoma
ECM	Extracellular matrix
HA	Hyaluronic acid
TAM	Tumor-associated
CAF	Cancer-associated fibroblast
TME	Tumor microenvironment
αSMA	Alpha smooth muscle actin
PSC	Pancreatic stellate cell
TGF β	Transforming growth factor β
IL	Interleukin
MMP	Matrix metalloproteinase
LIF	Leukemia inhibitory factor
GAS	Growth arrest-specific protein
FGF	Fibroblast growth factor
GDF	Growth differentiation factor
HGF	Hepatocyte growth factor
EMT	Epithelial–mesenchymal transition
MAPK	Mitogen-activated protein kinase
PI3K	Phosphatidylinositol 3-kinase
AKT	Protein kinase B
LOX	Lysyl oxidase
HAS	Hyaluronan synthase
EPR	Enhanced permeability and retention
PanIN	Pancreatic intraepithelial neoplasia
SCID	Severe combined immunodeficient
PDX	Patient-derived xenograft
PA	Polyacrylamide
PEG	Poly(ethylene glycol)
GelMA	Gelatin-methacryloyl
GelNB	Gelatin-norbornene
HPA	Hydroxyphenyl acetic acid
hiPSC	Human induced pluripotent stem cells
PNIPAM	Poly(N-isopropylacrylamide)
PEGDM	Polyethylene glycol dimethacrylate
PEGNB	Polyethylene glycol norbornene
PDMS	Polydimethylsiloxane
PVA	Polyvinyl alcohol
PAH	Poly (allylamine) hydrochloride
PAA	Poly (acrylic acid)
EDC	1-ethyl-3-(3-dimethylaminopropyl) carbo diimide
AFM	Atomic force microscope
YAP	Yes-associated protein
